# Autophagy-mediated nanomaterials for tumor therapy

**DOI:** 10.3389/fonc.2023.1194524

**Published:** 2023-12-15

**Authors:** Zijian Liao, Xiyu Liu, Dianfa Fan, Xingjun Sun, Zhikun Zhang, Pan Wu

**Affiliations:** ^1^ State Key Laboratory of Targeting Oncology, National Center for International Research of Bio-targeting Theranostics, Guangxi Key Laboratory of Bio-targeting Theranostics, Collaborative Innovation Center for Targeting Tumor Diagnosis and Therapy, Guangxi Medical University, Nanning, Guangxi, China; ^2^ School of Pharmacy, Guangxi Medical University, Nanning, Guangxi, China

**Keywords:** autophagy, signaling pathway, tumor therapy, nanomaterials, apoptosis

## Abstract

Autophagy is a lysosomal self-degradation pathway that plays an important protective role in maintaining intracellular environment. Deregulation of autophagy is related to several diseases, including cancer, infection, neurodegeneration, aging, and heart disease. In this review, we will summarize recent advances in autophagy-mediated nanomaterials for tumor therapy. Firstly, the autophagy signaling pathway for tumor therapy will be reviewed, including oxidative stress, mammalian target of rapamycin (mTOR) signaling and autophagy-associated genes pathway. Based on that, many autophagy-mediated nanomaterials have been developed and applied in tumor therapy. According to the different structure of nanomaterials, we will review and evaluate these autophagy-mediated nanomaterials’ therapeutic efficacy and potential clinical application.

## 1 Introduction

Autophagy is the process of isolation and degradation of cytoplasmic components by autophagosomes, a multistep lysosomal degradation pathway that supports nutrient cycling and metabolic adaptation. The primary role of autophagy is to label damaged organelles, cytoplasmic macromolecules and aggregated proteins and deliver them to lysosomes. Lysosomes are then degraded by lysosomal hydrolases to produce organic molecules such as amino acids, nucleotides, sugars and ATP, which are eventually recycled back into the cytoplasm (1). Autophagy is essential because it acts as a cytoprotective mechanism by technically avoiding the accumulation of damaged intracellular components, thus maintaining cellular homeostasis and energy metabolism and thus ensuring cell survival under conditions of stress and nutrient starvation (2). In terms of morphological and protein components involved in the core mechanisms, autophagy manifests itself as an extremely conserved intracellular process from yeast to mammals (3, 4). Depending on the pathway of action, autophagy is divided into three categories: macroautophagy, molecular chaperone-mediated autophagy, and microautophagy. Autophagy is usually referred to as macroautophagy (5, 6). Autophagy is associated with many physiological and pathological processes, such as neurodegenerative diseases, infections, and cancer.

### 1.1 Mechanism of autophagy

**Figure 1:**
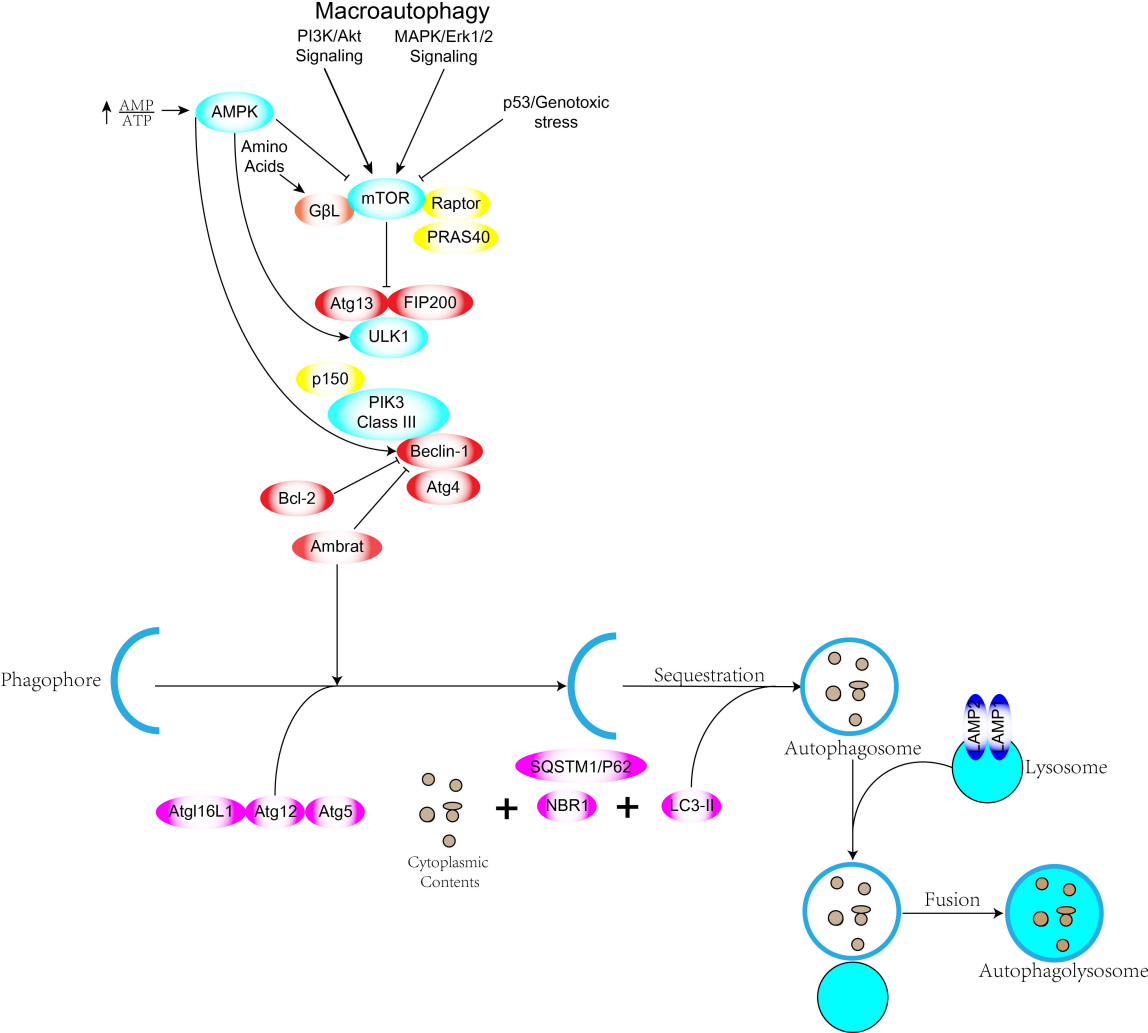
Schematic representation of the basic mechanism of macroautophagy.

Autophagy acts as a catalytic process leading to autophagic lysosomal degradation of major cytoplasmic contents (abnormal protein aggregates and excess or damaged organelles). Prior to autophagic lysosome assembly autophagic signaling is mediated by activation of the ULK complex consisting of ULK1 or ULK2, FIP200 and mATG13 (7). The ULK1 complex is the bridge *in vivo* that connects the upstream nutrient or energy receptors mTOR and AMPK with downstream autophagosome formation.ULK1 and ULK2 are highly phosphorylated and ULK1 has been reported to have Over forty phosphorylation sites have been reported (8). The ULK1 complex upon activation chimerizes to the membrane of the phagocytic vesicle and then other complexes are recruited to the site (9). Phosphorylated ULK1 has long been recognized as a key regulator of autophagy, and two kinases, AMPK and mTOR, have been found to catalyze the phosphorylation of ULK1, which plays a very important role in autophagy. Under starvation conditions, AMPK activates and mTOR inactivates, and the activated AMPK catalyzes phosphorylation of serines 317, 467, 555, 574, 637 and 777 of ULK1 to promote autophagy. Under nutrient-sufficient conditions AMPK inactivation, mTOR can bind to ULK1 serine at position 757 to inhibit ULK1-AMPK interaction, leading to inactivation of ULK1 and ultimately shutting down autophagic signaling (10, 11). mTOR kinase is an important regulatory molecule of autophagy (12) and activated mTOR (Akt and MAPK signaling) can inhibit autophagy, while negative regulation of mTOR (AMPK and p53 signaling) promotes autophagy (**Figure 1**). Three related serine/threonine kinases, UNC-51-like kinase -1, -2, and -3 (ULK1, ULK2, UKL3) (13), play a similar role to yeast Atg1 as downstream mTOR complexes. ulk1 and ulk2 are related to the mammalian homolog of the Atg gene product (mAtg13), the scaffolding protein FIP200 (homolog of yeast Atg17) to form a large class III PI3K complex. The complex includes hVps34, Beclin-1 (mammalian homolog of yeast Atg6), p150 (mammalian homolog of yeast Vps15) and Atg14-like protein (Atg14L or Barkor) or UVRAG (ultraviolet irradiation resistant associated gene), all required for autophagy induction (4, 14). Atg genes control autophagosome formation through the Atg12-Atg5 and LC3-II (Atg8-II) complexes (15). Atg12 is coupled to Atg5 in a ubiquitin-like reaction requiring Atg7 and Atg10 (E1 and E2-like enzymes, respectively). The Atg12-Atg5 linker then reacts non-covalently with Atg16 to form a larger complex. The C-terminus of LC3/Atg8 is cleaved by Atg4 protease to generate cytoplasmic LC3-I.LC3-I is also linked to phosphatidylethanolamine (PE) in a ubiquitin-like reaction that requires Atg7 and Atg3 (respectively E1 and E2-like enzymes). Finally, a lipid form of LC3, LC3-II (16), is formed and adsorbed on the autophagosomal membrane. There is both a positive and negative link between apoptosis and autophagy, and there is an extensive signaling “conversation” between the two processes. Autophagy has a pro-survival function when nutrients are lacking, but excessive autophagy leads to autophagic cell death, a morphologically distinct process from apoptosis. Some pro-apoptotic signals, such as TNF, TRAIL and FADD, can also induce autophagy.

### 1.2 Nanomaterials and autophagy

**Figure 2:**
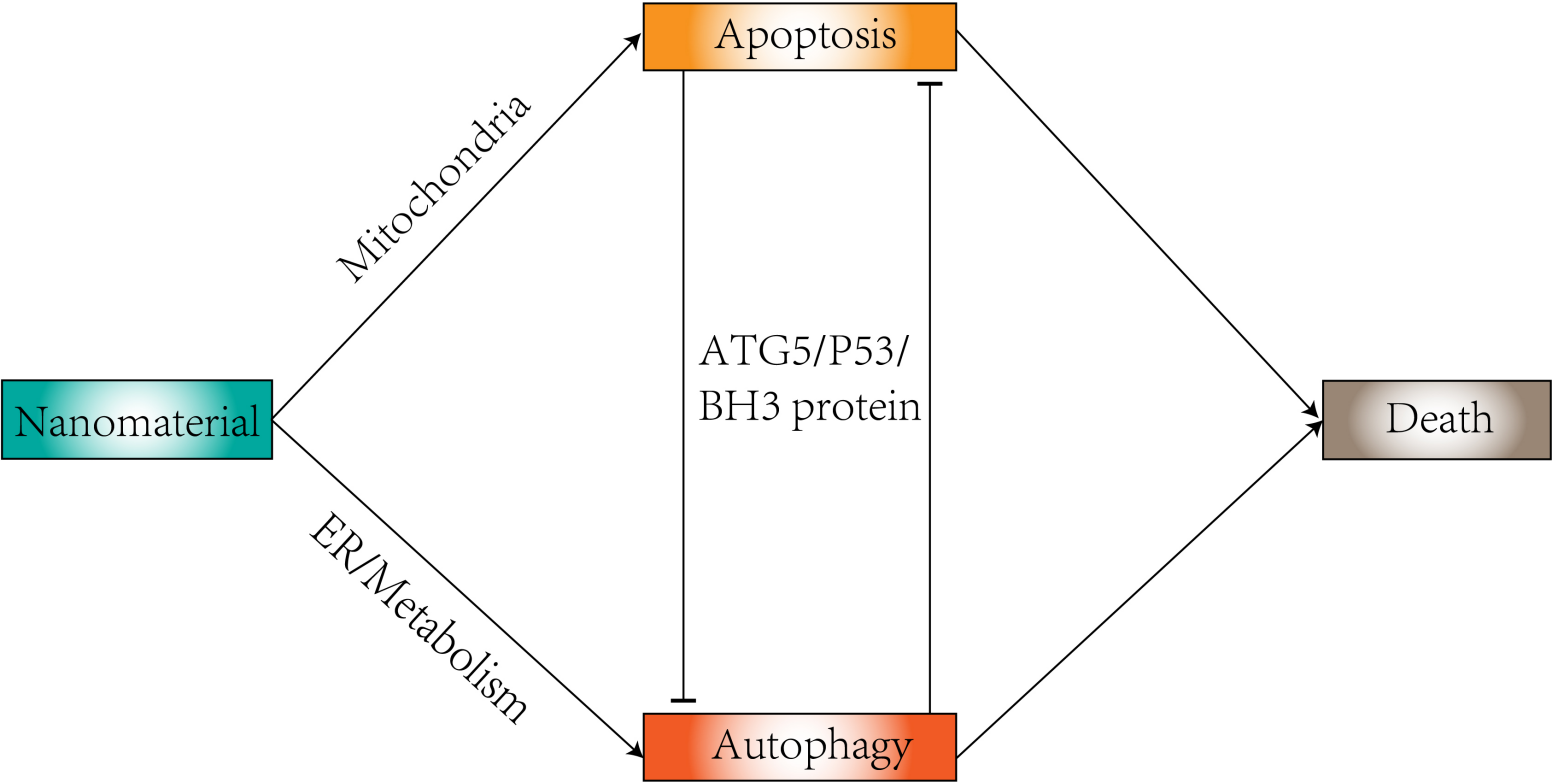
Relationship between autophagy and apoptosis induced by nanomaterials.

Nanomaterials are generally defined as particles in the size range between 10 and 100 nm (17), and their shape is directly related to the efficacy of biodistribution as a carrier and interaction with the target tissue (18). The most common are nanospheres and nanorods, but new nanocrystals have been developed. Their physicochemical characteristics (nanomaterial composition, concentration, size, surface charge, surface area, functionalization, dispersion state, protein corona, and cellular uptake) constitute a great advantage in autophagy (19). Compounds in autophagy modulation tend to have low bioavailability and can benefit from delivery using nanoparticles. Delivery using nanoparticles can benefit them (20). Nanoparticles have the ability to regulate autophagy (21, 22) (**Figure 2**). Changes in autophagy levels can lead to differences in cell biological behavior (23), which could be a potential therapeutic strategy to help in disease treatment. In tumor cells, the intervention of autophagy has been proposed as a target for cancer therapy. Many studies have revealed the role of autophagy generated by nanoparticles in tumor therapy based on their toxic effects (24), including gold nanoparticles, silver nanoparticles, and zinc oxide nanoparticles. Therapeutic interventions using nanoparticles to modulate autophagy can sensitize cancer cells to certain therapies (25). Nanoparticles can enter cells through the site of deposition and can also reach distant organs through a variety of mechanisms (26). Nanomaterials from many different compositions (e.g., metals, metal oxides, carbon, silica, and quantum dots) have shown cytotoxic effects in different biological systems (27–31). The cytotoxic potential of nanomaterials can be used to treat a wide range of diseases and conditions, as dysregulated pathways of apoptosis are a common feature of cancer, neurodegenerative diseases and neurological disorders (32, 33), and thus the apoptosis-modulating effects of nanomaterials are of great potential therapeutic value (34).

## 2 Nanomaterials and autophagy regulation mechanisms

**Figure 3:**
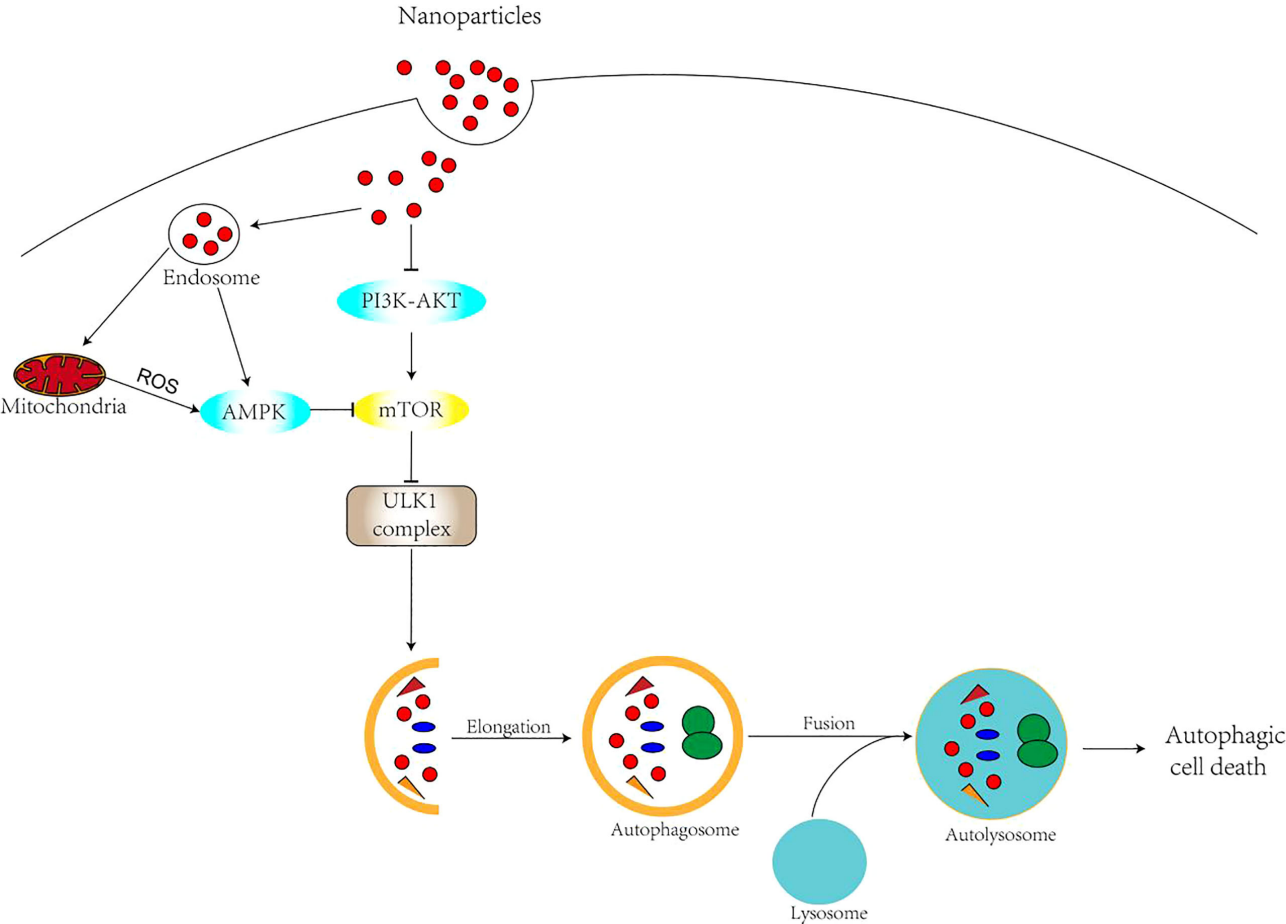
Mechanism of nanomaterials regulating autophagy.

Nanomaterials, as a novel regulator of autophagy, can affect autophagy through a variety of mechanisms. There are various types of nanomaterial-mediated autophagy. Oxidative stress has been widely recognized as one of the main causes of cytotoxicity of nanomaterials and is thought to play an important role in the regulation of autophagic processes (35, 36).There is a complex interaction between ROS and autophagy. With elevated levels of reactive oxygen species and H2O2, the AMPK pathway can be activated, thus inhibiting the mTOR pathway (37) (**Figure 3**). Meanwhile, ROS can directly affect the activity of ATG4. Oxidative stress leads to ATG4 oxidation and inactivation, which causes ATG8 lipidation and induction of autophagy (38, 39). There is also evidence that ROS increase Beclin1 (33) expression. In addition, degradation of nanomaterials in lysosomes can directly induce ROS (40). Nanomaterials can release redox-active metal ions, such as Fe2+ from gold-coated IONPS, which are involved in ROS generation (41). Lysosomes are a frequent target of nanomaterial autophagy, as most nanomaterials enter the cell by endocytosis. The accumulation of nanomaterials in lysosomes leads to the expansion of lysosomes and the release of histone proteases that accompany and sustain the increase of ROS and autophagy. On the one hand, autophagy is promoted as a cytoprotective mechanism to compensate for the lack of lysosomal degradation capacity (42). On the other hand, there is increasing evidence that nanomaterials cause lysosomal alkalinization, leading to lysosomal damage, affecting the fusion of lysosomes with autophagosomes, and ultimately leading to blocked autophagy. As novel regulators of autophagy, nanomaterials are mainly regulated through oxidative stress, direct regulation of autophagic signaling pathways and alteration of autophagy-related gene or protein expression levels (43, 44). Studies have shown that nanomaterials with smaller sizes (<1.4 nm) are more cytotoxic and induce necrosis, while nanoparticles larger than 1.4 nm usually induce apoptosis usually induce apoptosis (45, 46). Moreover, in addition to nanoparticle size its surface charge can also be a major factor in the mode of cell death induced. Related studies (47, 48) showed that charged gold nanoparticles induce apoptosis, while neutral nanoparticles trigger necrosis. In recent years, nanomaterial-induced autophagy has attracted increasing attention. Numerous studies have confirmed the potential of many types of nanomaterials in inducing cellular autophagy and apoptosis, including quantum dots (49–51), carbon-based nanomaterials (52–56), rare earth oxide nanocrystals (57), gold nanoparticles (58, 59), silver nanoparticles (35, 60), and silica nanoparticles (45, 61–63), among others.

### 2.1 Nanomaterials and oxidative stress

Oxidative stress is considered to be one of the main causes of nanoparticle-induced cytotoxicity and plays a very important role in the regulation of induced cellular autophagy (64). Oxidative stress induced by the massive production of ROS is often a mechanism of greater interest to researchers, as oxidative stress causes oxidative damage leading to a range of phenomena such as apoptosis, necrosis and ERS (65). Nanomaterial-induced autophagy production of ROS is often considered as an upstream signaling molecule to initiate the ERS-mediated apoptotic pathway. In tumor cells mitochondria-produced ROS’s play a very important role. On the one hand, nanomaterials can increase ROS production by interacting with mitochondria, and on the other hand, excited electrons on the surface of nanomaterials can also lead to an increase in intracellular ROS. ROS are involved in the regulation of the mTOR signaling pathway, activating or inhibiting mTORC1 activity in a dose- and time-dependent manner, and regulating autophagy and apoptosis. Lysosomes are considered as a regular target for nanoparticle-induced cytotoxicity and autophagy (66). The accumulation of nanoparticles in lysosomes leads to lysosomal swelling and release of histone proteases accompanied by increased levels of reactive oxygen species and autophagy. The accumulation of nanoparticles in lysosomes leads to lysosomal alkalinization and lysosomal damage, which can achieve selective lysosome induction in cancer cells potentially producing efficient anticancer effects and very low side effects (67). Related studies have shown that TiO2 NPS was able to increase ROS production in hepatocellular carcinoma cells, induce ERS and activate the PERK/ATF6/Bax signaling pathway, which inhibited the growth of hepatocellular carcinoma cells and the increase in tumor tissue volume (68). Studies have also noted that oxidative stress-mediated ERS is the main mechanism of neurotoxicity in human neuroblastoma (SH-SY5Y) cells (69). Given the relevant properties of nanoparticles in tumor cells, they hold great promise for application in the treatment of tumors through autophagy-induced generation of reactive oxygen species, broadening the therapeutic horizon of tumor patients.

### 2.2 Nanomaterials and regulation of autophagic signaling pathway

Nanoparticles can also directly interact with the mTOR signaling pathway to regulate autophagy and thus inhibit tumor cell growth, and in most cases, nano-induced autophagy accelerates cell death (70, 71). The induction of autophagy is strongly related to the physical properties of nanoparticles (e.g., dose, size, potential, physical properties, pH, etc.). Usually, high doses of nanomaterials are able to induce mTOR-mediated autophagy causing autophagic cell death (72). The ability of nanoparticles to generate autophagy in cells also lies in their physical shape and size (73). In BEAS-2B cells and two Si NP-treated groups, respectively, Nano-Si40 and Nano-Si60, induced PI3K/Akt/mTOR- controlled autophagy in a size-dependent manner. Upon elucidating the difference between MoS2 nanosheets with five-layer (2D NPs) and MoS2 nanosheets Upon elucidating the difference between MoS2 nanosheets with five-layer (2D NPs) and MoS2 nanosheets with 40-layer (3D NPs), they both activated the mTOR signaling pathway but the five-layer nanosheets, for the most part, only bound to the cell surface. proving that the cellular disturbance without NPs internalization also has medical and toxicological significance (74). In addition, pH sensitivity is one of the most effective factors in autophagy activation (72). Cationic PAMAM G3-activated autophagy is regulated by the Akt-TSC 2-mT0R pathway, but the anionic PAMAM G5.5 failed to elicit this response (75). Direct interaction of nanoparticles with the mTOR signaling pathway can inhibit tumor cell growth. During endocytosis, nanoparticles affect the recruitment/activation of PI3K/Akt in local regions of the cell membrane, thus altering the ability of Akt to activate mTORC1 In KP-SeNP nanomaterial-treated AGS cells, phosphorylation of PI3K/Akt/mTOR pathway markers and downstream targets is reduced, exerting anticancer effects through autophagy and apoptosis (76). In addition, there are related experiments verifying that Pt NCs regulate tumor cell apoptosis through PI3K-AKT-mTOR signaling that regulates autophagy (77). The role of autophagy and its related mechanisms in nanotoxicity cannot be ignored. It has been reported that due to their small size and other physicochemical properties (78), nanoparticles may cause damage to lysosomal and mitochondrial functions, inhibit autophagic processes through mTOR regulation, and even contribute to cytotoxicity. Therefore, for future human-friendly utilization of nanomaterials, physical properties such as concentration, size and surface charge of NPs should be carefully evaluated to present different roles in autophagy regulation, leading to normal cell survival or cancer cell death.

## 3 Application of nanomaterials in autophagy

In recent years, autophagy induced by nanomaterials has attracted increasing attention. Numerous studies have confirmed the potential of many types of nanomaterials to provide homeostasis of autophagy *in vivo*, and the different types of nanoparticles will be described in detail in this section (**Table 1**).

**Table 1:** Nanoparticles in the regulation of autophagy for cancer therapy.

Nanomaterials	Cancer type	Remarks	Refs
Au nanoparticles	Neuroglioma	Down-regulation of mTOR and PI3K/Akt Up-regulation of LC3-II and ERK Inducing autophagy	(79)
Silver nanomaterials	Bladder cancer	Triggering Akt and ERK signaling pathway Inducing autophagy	(80)
Iron nanomaterials	Breast cancer	PI3K/Akt expressions Up-regulation of LC3-II and ERK Inducing autophagy	(81)
Rare earth nanomaterials	Lung Cancer	Up-regulation of LC3 and Beclin1 expression Inducing autophagy	(82)
Zinc Nanomaterials	Breast cancer	Up-regulation of ATG5 Inducing autophagy	(83)
Graphene nanomaterials	Rectal Cancer	Triggering AMPK/mTOR/ULK-1signaling pathway Inducing autophagy	(84)

### 3.1 Gold nanomaterials

Gold nanoparticles possess many advantages such as simple preparation, stable physicochemical properties, controllable optical properties, and no apparent toxicity (85), and are the most widely used nanomaterials representing autophagy-induced nanomaterials. Indeed, an increasing number of reports demonstrate the clinical potential of gold nanoparticles as drug carriers (86), components of photothermal therapy (87), contrast factors (88), and therapeutic agents with significant cytotoxic activity (89) in the treatment of many cancers such as breast cancer (90), gastrointestinal tract cancer (91), lung cancer (92), and ovarian cancer (93). Lysosomal alkalinization and membrane permeabilization induced by gold nanoparticles, inhibition of autophagic flux can reduce the M2 polarization of TAMs and targeted autophagic intervention in antitumor immunotherapy (58). Mitochondria are the main targets of nanomaterial-induced oxidative stress, and once nanoparticles enter the mitochondria, pathways involving impaired electron transport chain, structural damage, NADPHase activation and mitochondrial membrane depolarization are initiated within the membrane. It has been shown (94) that the physicochemical properties of gold nanoparticles, especially the surface charge, strongly determine the mechanism of oxidative stress induction. Low-dose exposure to phytosynthetic gold nanoparticles combined with glutamine deprivation promotes cell death in cancer cells HeLa through oxidative stress-mediated mitochondrial dysfunction and G0/G1 cell cycle block (95). Properly modified gold nanoparticles have promising applications in cancer therapy.

### 3.2 Silver nanomaterials

Silver nanoparticles, with diameters ranging from 1 to 100 nm, are increasingly used in nanotechnology and nanomedicine applications and research because their smaller particle size is more readily absorbed by cells and has more opportunities to interact with cellular components. Silver nanoparticles enter cells into vesicles mainly through cytokinesis. Some related studies have shown that silver nanoparticles can induce autophagy in cells, which is associated with oxidative stress, manifested by an increase in reactive oxygen species (96). Mechanistic studies have shown that silver nanoparticles phosphorylation activates the PI3K/AKT/mTOR signaling pathway and induces the autophagic process. Silver nanoparticles can further trigger apoptosis by upregulating caspase-3 and Bax and downregulating Bcl-2 in cells (60). AgNPs impede autophagic flux by inhibiting the fusion of autophagosomes with lysosomes, thereby exacerbating AgNPs-induced neurotoxicity (97). In some undesirable malignancies, the radiosensitizing effect of silver nanoparticles on malignant gliomas was revealed to provide promising radiation therapy (98), and reactive oxygen species were associated with the autophagy-stimulating and radiosensitizing effects of silver nanoparticles (99). In human epithelial rectal cancer, silver nanoparticles killed cancer cells by inducing oxidative stress and DNA damage, induced a decrease in NFKB expression and an increase in IKB expression during autophagy, and autophagosome formation was accelerated by an increase in p53 and light chain 3 (LC3) II expression. In addition, inhibition of Akt and mTOR also played a key role in autophagy formation. Finally, autophagy overexpansion promotes apoptosis (100). Silver nanoparticles have been shown to induce nuclear translocation of transcription factor EB through a well-established mechanism involving dephosphorylation of transcription factor EB at serine 211 and serine 142, and nuclear translocation of transcription factor EB precedes autophagy stimulation (101), and even promotes increased expression levels of autophagy-essential genes through silver nanoparticle therapy, which positions TFEB as a potential target.

### 3.3 Iron nanomaterials

Iron nanoparticles are frequently used for various biomedical applications, and oxide nanoparticles, especially magnetic iron oxide nanoparticles in magnetite and magnetic hematite, are the most promising and popular iron oxide candidates because of their good chemical stability, magnetic responsiveness, and biocompatibility (102). The central mechanism of iron oxide induced autophagy is the Fenton reaction, which is summarized as chemodynamic therapy (CDT). Iron nanomaterials induce autophagy by reacting with endogenous H2O2 through Fenton or Fenton-like reactions, resulting in the *in situ* production of cytotoxic hydroxyl radicals to kill cancer cells (103). It has been shown that the effective inhibition of hepatocellular carcinoma growth by iron oxide in combination with other drugs achieves tumor suppression by enhancing intracellular iron retention inducing sustained reactive oxygen species (ROS) production through the Fenton reaction thereby inhibiting autophagosome and lysosome fusion (104). In addition, iron oxide nanoparticles can be used as a drug delivery vehicle with stronger autophagy-inducing effects in combination with paclitaxel, which increased the relative expression levels of Beclin1 and LC3II to LC3I, decreased the relative expression level of p62, increased intracellular iron ion concentration, activated ROS and lipid peroxidation, and downregulated the expression level of GPX4 protein. Exerted inhibitory effects on tumor cells by enhancing autophagy-dependent iron death pathway and lipid peroxidation (105).

### 3.4 Rare earth nanomaterials

Rare earth elements are natural components of the earth’s crust with unique chemical and physical properties, and in the biomedical field, their oxide nanoparticles have different biological functions *in vitro*, including protein adsorption, cellular uptake, antiviral activity, cell differentiation, oxidative stress, and neuroprotection, and are even considered as novel autophagy inducers. For rare earth elements, autophagy induction is a typical biological effect. It has been shown that novel lanthanides have high anticancer activity and induction of apoptosis and autophagy (82). Furthermore, variants of RE-1 peptide exhibit differentially reduced binding capacity and autophagy induction, which is thought to provide a pluripotent tool to modulate material-cell interactions to obtain desirable levels of autophagy. Glioblastoma is a heterogeneous disease with multiple genotypes (106) and is one of the most malignant of astrocytic tumors with a poor prognosis. Due to their unique electronic configuration, rare earth elements play a role in enhanced radiation therapy. It was shown that significant radiosensitization was observed in U-87 MG when incubated with Gd2O3,CeO2-Gd and Nd2O3:Si. Based on the radiosensitizing effect of Gd2O3 nanomaterials in U87 MG, their cell survival was significantly reduced by irradiation at 6 MV X-rays. These rare earth oxides do not produce any intrinsic cytotoxicity in the absence of irradiation and show high biocompatibility (107).

### 3.5 Zinc nanomaterials

Zinc oxide nanoparticles are one of the main nanomaterials used in the treatment of cancer. Studies have shown that impaired mitochondrial morphology and function in exposed cells triggers excessive ROS production, reduced Mito membrane potential, imbalance in Ca2+ homeostasis and release of cell death signaling molecules, ultimately leading to redox stress, apoptosis and inflammatory responses (108, 109). Therefore, ZnO nanoparticles are considered as a major breakthrough in tumor therapy. In hepatocellular carcinoma studies (110), it was found that zinc oxide nanoparticles promote autophagy and upregulate the expression of P53 and Caspase3. zinc oxide nanoparticles enter the cell by altering the mitochondrial membrane potential (111), increase the permeability of the outer membrane, and activate the expression of P53 and Caspase3. p53, an important oncogene, specifically inhibits Bcl-2 through transcriptional and translational expression (112).Caspase 3 activates apoptosis, promotes the release of cytochrome C, activates apoptosis protease activating factor 1, catabolizes DNA polymerase, and damages cellular DNA, thus reducing the proliferation and increasing apoptosis of cancer cells (113, 114). It has been demonstrated that ZnO nanoparticles with an average size of 20 nm are able to produce significant cytotoxicity in human ovarian cancer cells through the induction of intracellular ROS, through which they can directly affect the mechanical pathways of cell viability through apoptosis and autophagy, leading to mitochondrial disruption, as well as alterations in mitochondrial phosphate transporter (MPT) and function (115). In addition, ZnO nanoparticles-induced autophagy is closely related to chemoresistance in gastric cancer cells, and inhibition of autophagy can alleviate chemoresistance (116–118).

### 3.6 Graphene nanomaterials

Graphene oxide (GO) nanoparticles, as carbon-based nanocarriers, have the advantages of large surface area, good mechanical strength, and strong surface modification ability. It has a honeycomb structure with a high affinity for binding and its electrons are involved in the aro conjugation domain (119). These carbon-based nanocarriers have a high selectivity for tumor cells and can be used to deliver chemotherapeutic drugs in tumor therapy (120). In addition, graphene-based nanoparticles can inhibit cancer progression by providing photothermal therapy (121). Graphene oxide treatment leads to cytotoxicity, reactive oxygen species (ROS) production, apoptosis, autophagy and activation of AMPK/mTOR/ULK1 signaling pathway. Graphene oxide exerts anticancer effects on autophagy and apoptosis associated with colorectal cancer *via* ROS-dependent AMPK/mTOR/ULK-1 pathway. Related studies have shown that reduced graphene oxide stimulates autophagy and cell cycle arrest thereby inducing apoptotic death of cancer cells (122). By stimulating autophagy, GO nanocarriers can promote the sensitivity of cancer cells to chemotherapy. However, by impairing autophagic flux, GO nanoparticles can reduce cell survival and enhance inflammation. Similarly, graphene oxide nanomaterials can increase ROS production and induce DNA damage, thereby sensitizing cancer cells to apoptosis. Molecular pathways, such as ATG, MAPK, JNK and Akt, can be modulated by graphene oxide nanomaterials leading to effects on autophagy and apoptosis (53).

## 4 Conclusion

An increasing number of studies have shown that nanomaterial-induced autophagy plays an important role in the pathogenesis of tumor diseases and that different materials induce different phenomena of autophagy. The potential mechanisms of autophagy regulation by nanoparticles even with the same nanomaterial but with different sizes, shapes and surface modifications differ, which provides multiple models for studying autophagy. The autophagy regulation by nanomaterials is influenced by many factors such as material composition, particle size, shape, surface modification, dose, treatment time, synthesis method and cell line. Therefore, the regulatory outcome of nanoparticle-mediated autophagy varies in different situations. The extent and mechanisms of autophagy induction by various nanomaterials lack relatively uniform criteria. Whether as drug carriers or drugs, nanomaterials have shown good clinical diagnostic and therapeutic effects. Notably, a large number of nanomedicines have been proposed as research tools for the diagnosis or treatment of cancer. There is growing evidence that various types of nanomaterials can modulate the autophagic process of cells and thus induce apoptosis. The regulation of cellular autophagy by nanomaterials has received significant attention in cancer therapy. Selective induction of autophagy-mediated chemosensitization by various nanomaterials in cancer cells can be very beneficial for cancer therapy. With the in-depth study of various mechanisms of nanomaterials, it is believed that nanomaterials will provide more possibilities for clinical diagnosis and treatment.

## Author contributions

PW, ZZ: Conceptualization, Methodology. ZL, XL: Writing- Original draft preparation. DF, XS: Software. LP: draw designs. All authors contributed to the article and approved the submitted version.
